# Exploring Patients' Intentions for Continuous Usage of mHealth Services: Elaboration-Likelihood Perspective Study

**DOI:** 10.2196/17258

**Published:** 2020-04-06

**Authors:** Xitong Guo, Shuqing Chen, Xiaofei Zhang, Xiaofeng Ju, Xifu Wang

**Affiliations:** 1 Guangzhou First People’s Hospital School of Medicine South China University of Technology Guangzhou China; 2 School of Management Harbin Institute of Technology Harbin China; 3 Business School Nankai University Tianjin China

**Keywords:** mHealth services, health consciousness, elaboration-likelihood model, health behavior, patients’ continuous usage

## Abstract

**Background:**

With the increasingly rapid development of Web 2.0 technologies, the application of mobile health (mHealth) care in the field of health management has become popular. Accordingly, patients are able to access consulting services and effective health information online without temporal and geographical constraints. The elaboration-likelihood model (ELM) is a dual-process persuasion theory that describes the change of attitudes and behavior.

**Objective:**

In this study, we drew on the ELM to investigate patients’ continuous usage intentions regarding mHealth services. In addition, we further examined which route—central or peripheral—has a stronger impact on a patient’s usage of health care management.

**Methods:**

To meet these objectives, five hypotheses were developed and empirically validated using a field survey to test the direct and indirect effects, via attitude, of the two routes on continuous usage intention.

**Results:**

We found that patients’ perceived mHealth information quality and perceived mHealth system quality had a positive effect on their personal attitudes. The results revealed that social media influence had a positive effect on a patient’s attitude toward mHealth services. In particular, our findings suggest that a patient’s health consciousness has a positive effect on the relationship between social media influence and attitude.

**Conclusions:**

This study contributes to the mHealth services literature by introducing the ELM as a referent theory for research, as well as by specifying the moderating role of health consciousness. For practitioners, this study introduces influence processes as policy tools that managers can employ to motivate the uptake of mHealth services within their organizations.

## Introduction

### Background

Advances in Web 2.0 technology have resulted in the application of mobile health (mHealth) care to the field of health management with increasing popularity. According to the Mobile Medical Survey Report of 2016, there were about 90,000 mHealth apps on the iOS platform in the United States in 2015. Compared with that of 2013, the growth rate was 106%. In October 2015, the China State Council implemented the Internet Plus policy, which enhanced the status of internet technology in health care. In October 2016, the outline of the Healthy China 2020 plan was proposed to establish a healthy concept to realize the health care needs of the entire nation. In January 2017, the Chinese government proposed the 13th Five-Year Plan for Health Promotion and Work and established the activities and plans for health promotion. In February 2017, the Chinese government proposed a long-term plan for the prevention and treatment of chronic diseases in China, which fully expressed the concept of the management of chronic diseases in daily lives. Studies have shown that health management apps have a positive impact on the improvement of personal health [1,2]. However, realistically, in all health management apps, the actual usage rate is rather low and the effects of health management are not obvious [3]. In our research, we ask the following questions: What are the reasons for the low utilization of health management apps? What factors affect users’ low compliance with mHealth apps and health behavior changes?

Prior literature suggests that the availability and ease of the use of health management apps play a crucial role in a patient’s choice of whether or not to use an app [4]. In 2009, Sykes et al [5] indicated that the professional support provided by a health management app to patients exerts a positive impact on the use of the app. Another study found that the patient’s personal intrinsic motivation has a positive effect on his or her health behavior changes [6]. Chen also explored the attitude toward organic food among the Taiwanese, which is related to health consciousness, environmental attitudes, and the mediating effects of a healthy lifestyle [7]. In fact, he found that health consciousness and the environment are the two most important motives for purchasing organic food. In addition, healthy lifestyle acts as a positive mediating effect between health consciousness and environmental attitudes, as well as the consumer’s attitudes toward organic foods [7]. Most of the previous studies have shown that system and information service quality, one’s motivation and ability, as well as social influence could affect the usage of apps. However, there is no clear evidence on the types of information that are most effective in influencing a patient’s continuous usage intention and whether these influences are temporally persistent.

Bhattacherjee and Sanford found that the influence processes for information technology (IT) acceptance are based on an elaboration-likelihood model (ELM) [8]. This theory enhanced our understanding of influence processes in IT acceptance based on the ELM. Indeed, the ELM includes two types of routes: central and peripheral routes are based on the type of information processed by a given user. The ELM also explains how one route may cause more influence than the other and how each route can have a long-term impact on users’ behavior changes [8,9]. Therefore, in this study, we conducted in-depth research on the central route (ie, intrinsic motivation) and peripheral route (ie, external motivation) of the ELM as well as on health awareness through multifactor perspectives at the individual, organizational, and social levels. We also determined which route—the central or the peripheral route—has a stronger impact on a patient’s use of health management apps. In particular, we planned to answer to the following research questions:

1. Question 1: What are the factors that affect a patient’s continuous usage intention of mHealth services?
2. Question 2: How does health consciousness affect a patient’s continuous usage intention of mHealth services?
3. Question 3: Which factor has a stronger effect on a patient’s continuous usage intention of mHealth services?

Seeking the answers to these three questions can enable us to better understand the factors that affect patients’ continuous usage of mHealth.

To meet the aforementioned objectives, a survey was conducted to test the main and mediated effects of mHealth services on a patient’s continuous usage intention. Our participants were users of mHealth apps in China. We tested the proposed model using structural equation modeling (SEM) with a partial least squares (PLS) estimation.

This study provides several theoretical contributions. First, we expanded the ELM and applied it to the health sector. Second, we explored the central and peripheral routes of the ELM, each with a different motivation and ability regarding the effects of patients’ continuous usage intention. Third, we redefined the cognitive-attitude-change process based on the original ELM.

Moreover, this study also contributes to the practical understanding of mHealth services in several important ways. First, as a result of the verification of our model, hospital administrators are provided with a theoretical basis, which enables them to better understand a patient’s psychological characteristics and accordingly construct a more comprehensive informatization process. Second, our model can provide support for health management entrepreneurs in terms of the design of health management apps so that they can clearly identify the problems and directions facing them. Third, the patients themselves can also gain a clear understanding of their inner tendencies, so that they can identify health management systems that best suit their personal characteristics through many health management apps and, thus, better manage their own health problems.

The rest of this paper proceeds as follows. In the next section (Theoretical Background), we present a brief overview of prior research and identify gaps in health behavior through mHealth service management. In the following section (Research Model and Hypotheses), we describe the key constructs and relationships in the ELM and present five hypotheses. The proposed hypotheses are tested using a survey based on a dataset of the app users of the mHealth service. This is followed by a discussion of our findings and their implications for information service research and practice. We end with some conclusions.

### Theoretical Background

#### mHealth Services

mHealth services, as a new health management approach to break time and space constraints, can employ communication technology and mobile devices to provide personalized health management, real-time information tracking, and other services for patients [10]. They can also provide services for all patients without temporal and geographical constraints. Compared with traditional health management approaches, mHealth services enable users to access unlimited data and more effective disease control approaches. With an inflow of medical capital, policy support, and technology development, mHealth services have undergone rapid development. According to the Mobile Medical Survey Report of 2017 [11], the stickiness of mHealth app users has generally been seen as being on the rise since 2016, and mHealth services are gradually becoming one of the ways for patients to access health management. Because of their mobility, traceability, and high levels of interaction, mHealth apps provide solutions for effective health management. Thus, mHealth apps alleviate the health care pressures and economic burdens of patients; improve the well-being of individuals, families, and society; and achieve the maximization of medical values. On the one hand, mHealth services provide effective information for patients, which could enable them to perform effective health self-management without the constraints of time and location. On the other hand, mHealth services provide effective communication channels for patients, family members, and doctors and thereby enable patients to receive emotional and professional support from family members and doctors.

#### The Elaboration-Likelihood Model

**Figure 1:**
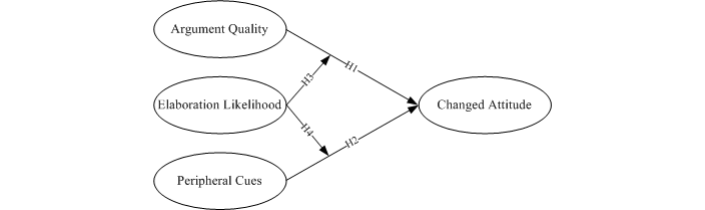
Original model for the elaboration-likelihood model. H1: Hypothesis 1; H2: Hypothesis 2; H3: Hypothesis 3; H4: Hypothesis 4.

The ELM was developed by Petty and Cacioppo in 1986 [9]. It is a dual-process persuasion theory that describes the change of attitudes. The ELM mainly includes the central route and the peripheral route to persuasion. The central route to persuasion—where elaboration is high—is probably due to the careful and thoughtful consideration of the true value of the information provided by a person to support advocacy. The central route involves a high level of message elaboration, where the individual receiving the message generates a large amount of cognitions about the arguments. The result of changing attitudes will be relatively long-lasting, resistant, and predictive of behavior. In contrast, the peripheral route to persuasion—where elaboration is low—is caused by a person’s connection with positive or negative clues in the stimulus or by simple reasoning about the merits of the claimed position. The prompts that an individual receives in a peripheral route are usually independent of the logical quality of the stimulus. These tips will relate to factors such as the credibility or appeal of the source or the quality of the message. The likelihood of elaboration will be determined by an individual’s motivation and ability to evaluate the argument being presented. The central and peripheral routes of attitude change are typically operationalized in ELM research using the argument quality and peripheral cues constructs, respectively, as shown in Figure 1.

Prior scholars have widely implied that the ELM applies in e-commerce, advertising, and other fields. Cheung et al examined four information cues to evaluate the credibility of online reviews drawing on the ELM [12]. Ho and Bodoff also integrated the ELM and consumer search theory to illustrate how depth and breadth influence a user’s attitude toward a personalization agent and item selection [13]. In addition, Cao et al investigated patients’ selection decisions based on the ELM and the service quality theory; they also examined the moderating effects of disease risk and disease knowledge on patients’ consulting intention [14]. Although the above studies demonstrated the effectiveness of the ELM in user behavior, user purchase, and patients’ consulting intention in the online health community, to the best of our knowledge, there is no study involving patients’ continuous usage intention toward mHealth services.

### Research Model and Hypotheses

#### Overview

**Figure 2:**
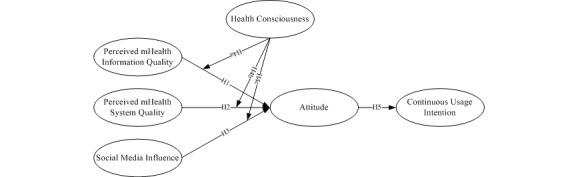
The conceptual research model based on the elaboration-likelihood model. H1: Hypothesis 1; H2: Hypothesis 2; H3: Hypothesis 3; H4a: Hypothesis 4a; H4b: Hypothesis 4b; H4c: Hypothesis 4c; H5: Hypothesis 5.

Based on the aforementioned theoretical foundations, our research model is proposed as shown in Figure 2. In the model, the central routes (ie, perceived mobile information quality and perceived mobile system quality) and the peripheral route (ie, social media influences) are considered as antecedents of the model; to these, we add the moderating effect (ie, health consciousness) on the attitude, which in turn influences a patient’s continuous usage intention.

#### Perceived mHealth Information and System Quality Based on a Patient’s Attitudes

Previous studies have shown that the quality of e-services has a positive effect on customers’ attitudes [15]. Wixom and Todd studied the impact of service quality on user satisfaction and divided service quality into information quality and system quality in the e-service context [16]. Cao et al revealed that service quality and e-word-of-mouth both had positive effects on patients’ selection decisions [14]. Similar to e-service, mHealth service, as a new service platform and channel, has attracted considerable attention. Information quality and design standards in mHealth service apps can have an impact on patients’ perceptions.

Thus, we hypothesize the following:

1. Hypothesis 1: Patients perceive that the information quality of an mHealth service has a positive effect on their personal attitudes.
2. Hypothesis 2: Patients perceive that the quality of the systems of an mHealth service has a positive effect on their personal attitudes.

#### Social Media Influence on Patients’ Attitudes

In addition to focusing on app-provided information, patients will also take into consideration external information, which influences their decision making. The prior study proposed that entertainment, sociality, information, and trust positively influence WeChat users’ attitudes and users’ trust [17]. Treviño et al put forward that medium symbolism, message equivocality, distance between message partners, perceived media richness, number of message recipients, and perceived message recipients’ attitudes are the factors that affect the user’s media choices [18]. Erkan and Evans also found that the key factors of social media that influence consumers’ purchase intentions were quality, credibility, usefulness and adoption of information, need for information, and attitude toward information [19]. Therefore, they take into consideration the peripheral route, which includes the persuasive effects of information conveyed by social media on patients.

Thus, we hypothesize the following: Social media influence has a positive impact on a patient’s attitude (Hypothesis 3).

#### The Moderation Effects of Health Consciousness

In addition to the effects of the central and the peripheral routes, an individual’s health consciousness is related to his or her ability to receive information. Studies have shown that the higher the level of a patient’s health consciousness, the more concerned that patient would be about his or her health [20]. Scholars have shown that people with high health-literacy rates have quick access to information and strong judgment [21]. In addition, a previous study has illustrated that social cognitive factors and all the perceived social influence variables significantly improved eHealth literacy [22]. Hence, we propose that personal health consciousness plays a moderating role in the ELM.

Thus, we hypothesize the following:

1. Hypothesis 4a: A patient’s health consciousness has a positive moderating effect on his or her relationship between the perceived information quality and the contents and attitudes of mHealth services.
2. Hypothesis 4b: A patient’s health consciousness has a positive moderating effect on the relationship between the perceived service quality and the systems and attitudes of mHealth services.
3. Hypothesis 4c: A patient’s health consciousness has a negative moderating effect on the relationship between social media influence and attitudes.

#### The Attitudes and Continuous Usage Intention of Patients

Prior studies have inferred that a patient’s attitude has a positive effect on his or her usage intention. Thus, we hypothesize the following: A patient’s attitude has a positive effect on his or her continuous usage intention (Hypothesis 5). The descriptions and definitions of the constructs of our hypotheses are presented in Table 1.

**Table 1:** The descriptions and definitions of the constructs.

Construct	Description
Perceived mHealth information quality	A patient’s perception of the quality of information provided by the mHealth management apps
Perceived mHealth system quality	A patient’s perception of the quality of the system provided by the mHealth management apps
Social media influence	The persuasive effects of the information conveyed by social media on a patient’s attitude to the mHealth management apps: this is not a component of the app itself
Health consciousness	A patient’s attention to his or her personal health issues or the motivation to protect them
Attitude	A patient’s attitude toward the mHealth management apps
Continuous usage intention	The willingness of patients to continue to use their health management apps

## Methods

### Overview

To test our model, we conducted a survey. This method was chosen because it is a way to access perceived information quality, perceived system quality, social media influence, attitude, and continuous usage intention; as well, it can enhance the broader applications of the research findings [23].

### Sample of the Normal Distribution Test

Bentler and Chou stressed that data can be structured for analysis on the premise that the sample dataset satisfies the normal distribution [24]. Although the results of our study indicate that the software program SmartPLS (SmartPLS GmbH) is suitable for nonnormal distribution, the results are better under conditions of a normal distribution. The condition by which the sample data satisfies a normal distribution occurs after the descriptive test of each constructed measurement item index, when the absolute value of the skewness is less than 2, and while the absolute value of the kurtosis is less than 5.0. We used SPSS Statistics for Windows, version 23.0 (IBM Corp), for statistical analysis of our sample data. The main output indicators were measured (ie, mean, SD, minimum, maximum, skewness, and kurtosis). The specific results are shown in Table 2.

We can perceive from this table that the largest value of skewness of 1.433 is less than 2, while the largest value of kurtosis of 2.269 is less than 5. Therefore, our survey data basically conforms to the above normal distribution and can be used for further structural equation analysis.

**Table 2:** Sample of the normal distribution test.

Item	Sample size	Min^a^	Max^b^	Mean (SD)	Skewness	Kurtosis
PIQ^c^1	255	1	7	5.52 (0.917)	-0.759	1.723
PIQ2	255	1	7	5.42 (1.123)	-0.589	0.457
PIQ3	255	1	7	5.54 (1.086)	-0.777	1.203
PIQ4	255	1	7	5.57 (1.154)	-0.958	1.335
PIQ5	255	2	7	5.13 (1.077)	-0.527	0.399
PSQ^d^1	255	2	7	5.39 (1.013)	-0.586	0.442
PSQ2	255	1	7	5.35 (1.151)	-0.644	0.483
PSQ3	255	1	7	5.37 (1.082)	-0.822	1.239
PSQ4	255	2	7	5.44 (1.138)	-0.697	0.396
PSQ5	255	1	7	5.06 (1.247)	-0.402	0.071
SMI^e^1	255	1	7	4.80 (1.469)	-0.663	-0.031
SMI2	255	1	7	4.85 (1.466)	-0.533	-0.125
SMI3	255	1	7	4.92 (1.456)	-0.642	-0.137
SMI4	255	1	7	4.93 (1.444)	-0.626	-0.207
HC^f^1	255	2	7	5.45 (1.244)	-0.953	0.612
HC2	255	3	7	5.73 (1.170)	-0.671	-0.432
HC3	255	1	7	5.83 (1.024)	-0.956	1.492
HC4	255	1	7	5.00 (1.290)	-0.639	0.325
HC5	255	2	7	5.79 (1.126)	-1.051	0.955
HC6	255	2	7	5.92 (1.145)	-0.947	0.475
AT^g^1	255	2	7	5.54 (1.071)	-0.612	0.244
AT2	255	2	7	5.46 (1.064)	-0.683	0.552
AT3	255	1	7	5.14 (1.289)	-0.692	0.215
AT4	255	1	7	5.35 (1.213)	-0.949	1.191
AT5	255	2	7	5.56 (1.175)	-0.832	0.617
CIU^h^1	255	1	7	5.87 (1.267)	-1.364	1.996
CIU2	255	1	7	5.69 (1.367)	-1.169	0.982
CIU3	255	1	7	5.76 (1.307)	-1.433	2.269
CIU4	255	1	7	5.71 (1.305)	-1.302	1.868

^a^Min: minimum.

^b^Max: maximum.

^c^PIQ: perceived information quality.

^d^PSQ: perceived system quality.

^e^SMI: social media influence.

^f^HC: health consciousness.

^g^AT: attitude.

^h^CIU: continuous usage intention.

### Study Setting and Demographic Details of Participants

The survey questionnaire was distributed to the participants via WJX [25], a questionnaire website that is the oldest and most-used online survey software and serves as an examination and voting platform in China. Since its launch in 2006, users have posted more than 28.22 million questionnaires and have collected more than 1.88 billion responses while maintaining a growth rate of more than 100% yearly. The platform’s users have covered more than 90% of universities and research institutions in China, and the platform is a well-known portal for questionnaires, examinations, and voting systems that are trusted by leading companies in various industries.

The survey was posted on the questionnaire website. The objectives of the survey and the requirements for the recruitment of our participants were introduced at the beginning of the survey, and each participant would receive an incentive of RMB 15 (approximately US $2.19). The participants were from all over China and were users of mHealth management apps, such as Haodf, XYWY, or Guahao, which are large-scale mHealth apps in China. The data collection process was divided into two phases. In the first phase, a pilot analysis was conducted and the measurement model (eg, the reliability, validity, common method bias, and multicollinearity of the constructs) was assessed to ensure its appropriateness. In the second phase, we collected the data. A total of 262 questionnaires were distributed. Of these, 255 contained acceptable responses, yielding a response rate of 97.3%. Of these respondents, 55.3% (141/255) were male and 79.6% (203/255) were under the age of 40 years. Most of the participants were employed (241/255, 94.5%). Among the participants, 67.1% (171/255) had graduated with a bachelor’s degree.

The demographics of the respondents are shown in Table 3. A 7-point Likert scale, ranging from 1 (*strongly disagree*) to 7 (*strongly agree*), was used in our research. The scores indicate different levels of health consciousness for the two groups in our sample and we believe this approach was appropriate for our research.

**Table 3:** Demographics of the respondents.

Measure and category		Value (N=255), n (%)
**Age in years**		
	<40	203 (79.6)
	40-50	45 (17.6)
	51-60	6 (2.4)
	>60	1 (0.4)
**Gender**		
	Female	114 (44.7)
	Male	141 (55.3)
**Education**		
	Junior middle school or below	1 (0.4)
	High school	4 (1.6)
	Special secondary school	14 (5.5)
	Junior college	38 (14.9)
	Bachelor’s degree	171 (67.1)
	Master’s degree or above	27 (10.6)
**Work status**		
	Working	241 (94.5)
	Retired	14 (5.5)

### Measurements

The measurement of the majority of constructs was adopted from prior relevant studies (see Multimedia Appendix 1). Slight modifications were necessary to make the text suitable for the research context, and all measures used a 7-point Likert scale. Sustained participation was measured using the instrument of continuous usage intention that was adopted from Bhattacherjee and Sanford [8] and from Petty and Cacioppo [9]. Perceived mHealth information quality was measured using methods by Park et al [26] and by Bhattacherjee and Sanford [8]. Perceived mHealth system quality was measured using methods by Cheong and Park [27] and by Brady and Cronin [28]. Social media influence was measured using methods by Keery et al [29]. Attitude was measured using methods by Kim [30] and by Yang and Yoo [31]. The six items used to measure health consciousness were adopted from Mai and Eisenberg [32] and from Chen [7].

To enhance the validity of the measures of the constructs, we followed Moore and Benbasat [33] when adapting the measurements for our study. All the measures were adapted from prior research along with their sources (see Multimedia Appendix 1). Other control variables, such as age, gender, education, and work experience, were measured using a single-item measure. To verify the adapted survey items, individual meetings were held with university colleagues and postgraduate students to discuss the following: (1) the appropriateness of the questionnaire items, (2) the possibility of ambiguity in the questionnaire items, and (3) the appearance and layout of the survey instrument. Based on the feedback received, a revised questionnaire was developed. This was then sent to the same individuals for a second review. A minor revision was made based on their further suggestions.

### Data Analysis and Results

#### Analysis Strategy

We tested the hypothesized relationships among the constructs using SEM with the software program SmartPLS 3.0 (SmartPLS GmbH) [12,34]. A two-stage analytical procedure was used to analyze the data [35]. First, we assessed the validity of the measurement model and then examined its structure. PLS is a powerful component-based method that has been widely used in prior research [36]. It does not require multivariate normal distribution and has a minimal sample size requirement as compared to other SEM packages (eg, linear structural relationships [LISREL], analysis of moment structure [AMOS], and equation structural program [EQS]) [34,37,38]. In addition, it simultaneously estimates the structural and measurement models [36]. As our sample size is relatively small, we chose PLS to analyze the data [34]. Specifically, SmartPLS was used to conduct the data analysis [39].

#### Measurement Model

As essential prerequisites for achieving valid results, the reliability, convergent validity, and discriminant validity of the measurement model were assessed. Reliability was assessed by applying the Cronbach alpha and composite test for reliability. As shown in Table 4, Cronbach alpha values range from .718 to .881 and the composite reliability values range from .797 to .918, both of which exceed the recommended value of .70, thus confirming their reliability.

Table 5 shows that most of the item loadings were above .70, thus indicating convergent validity [40]. Moreover, the factor loadings of each construct were much greater than the cross-loadings on other constructs, and correlations of the constructs were much smaller than the square root of the *average variance extracted* of each construct, thus indicating discriminant validity [41], as shown as Table 6.

**Table 4:** The results of confirmatory factor analysis: construct reliability and validity (N=255).

Indicator (abbreviation)	Cronbach alpha	rho_A	Composite reliability	Average variance extracted
Attitude (AT)	.774	.777	.855	.797
Continuous usage intention (CUI)	.881	.881	.918	.737
Health consciousness (HC)	.795	.701	.811	.717
Perceived information quality (PIQ)	.729	.734	.830	.751
Perceived system quality (PSQ)	.718	.720	.797	.767
Social media influence (SMI)	.866	.868	.909	.714

**Table 5:** Item loadings and cross-loadings for each construct (N=255).

Construct (abbreviation) and items		Item loadings and cross-loadings					
		AT	CUI	HC	PIQ	PSQ	SMI
**Attitude (AT)**							
	AT1	.789	.471	.394	.402	.290	.407
	AT3	.711	.387	.363	.309	.347	.299
	AT4	.791	.453	.306	.350	.386	.336
	AT5	.796	.427	.311	.330	.333	.274
**Continuous usage intention (CUI)**							
	CUI1	.460	.862	.295	.334	.222	.290
	CUI2	.504	.872	.285	.352	.248	.198
	CUI3	.491	.860	.271	.391	.220	.244
	CUI4	.481	.839	.232	.350	.228	.311
**Health consciousness (HC)**							
	HC2	.298	.277	.709	.223	.148	.141
	HC3	.255	.172	.723	.159	.129	.094
	HC4	.398	.203	.736	.194	.241	.189
	HC5	.299	.257	.709	.206	.120	.181
**Perceived information quality (PIQ)**							
	PIQ1	.364	.331	.253	.777	.406	.200
	PIQ2	.335	.327	.220	.716	.464	.218
	PIQ3	.279	.237	.114	.714	.442	.166
	PIQ4	.356	.328	.205	.760	.428	.223
**Perceived system quality (PSQ)**							
	PSQ1	.302	.210	.180	.437	.741	.201
	PSQ2	.346	.257	.207	.483	.771	.167
	PSQ5	.337	.136	.137	.398	.746	.286
**Social media influence (SMI)**							
	SMI1	.350	.263	.146	.261	.275	.857
	SMI2	.342	.199	.179	.107	.227	.829
	SMI3	.373	.296	.185	.251	.203	.825
	SMI4	.385	.263	.219	.296	.273	.868

**Table 6:** Correlation matrix (N=255).

Construct (abbreviation)	Correlation					
	AT	CUI	HC	PIQ	PSQ	SMI
Attitude (AT)	.772					
Continuous usage intention (CIU)	.564	.858				
Health consciousness (HC)	.446	.315	.719			
Perceived information quality (PIQ)	.453	.416	.273	.742		
Perceived system quality (PSQ)	.438	.268	.232	.584	.753	
Social media influence (SMI)	.430	.303	.217	.274	.290	.845

#### Common-Method Bias Testing

As our data were collected from self-report surveys from the participants, common-method bias may threaten the validity of the results [42]. We used a modified-marker variable analysis to test the common-method bias in our model following Rönkkö and Ylitalo [43]; three items in our dataset, which have low correlation with the items in this study, were used to measure the marker variable. Next, the marker variable was incorporated into the model with its impacts on the endogenous variables. The results showed that the marker variable had no significant impacts on perceived mHealth information quality, perceived mHealth system quality, social media influence, attitude, and continuous usage intention; as well, hypothesized relationships had no significant differences regardless of whether or not the marker variable was introduced into the model. The results indicate that common-method bias had little impact on the results of our study [43].

#### Structural Model

**Figure 3:**
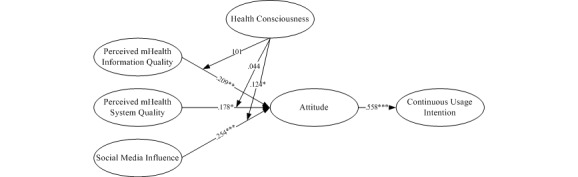
The conceptual research model based on the elaboration-likelihood model and results of the partial least squares analysis. *P<.05; **P<.01; ***P<.001.

An analysis of the structural model formed the second stage in the SEM. The significance of each path coefficient was calculated by bootstrapping with 5000 samples using the replacement method. In addition, SmartPLS supports the function of testing, whether the moderator of a relationship is significant or not in the model. We were able to choose this factor as a moderator in the tool directly and the test of the entire model would include the analysis of the moderator. In Figure 3, we present the results of the structural model. The model explained 32.9% of the variance in intention to continue usage and 43.4% of the variance in attitude. Hypothesis 1 posits that perceived information quality influences the attitude toward continuous usage intention. From Figure 3, it can be observed that the path coefficient is .209 (*P*=.003), thus supporting Hypothesis 1. Moreover, Hypothesis 2, which states that the perceived system quality affects the attitude toward continuous usage intention, is also confirmed (beta=.178, *P=*.03). The positive effect of social media influence on the attitude toward continuous usage intention is also supported (beta=.254, *P*<.001), thereby confirming Hypothesis 3. Furthermore, the effects of the control variables—age, gender, and education—on the continuous usage intention have been found to be insignificant.

We also tested the moderation effects of health consciousness between perceived information quality, perceived system quality, social media influence, and attitude. Following prior research, three steps were required to test the mediation effects [44,45]. In Step 1, we treated perceived information quality, perceived system quality, and social media influence as independent variables and attitude as the dependent variable; we found a significant relationship between them: beta=.250, *P*<.001; beta=.205, *P*=.02; and beta=.302, *P*<.001, respectively. In Step 2, we built a model that added health consciousness as an independent variable and attitude as the dependent variable, after which we found a significant effect: beta=.196, *P=*.003; beta=.179, *P=*.03; beta=.261, *P*<.001; and beta=.294, *P*<.001, respectively. In Step 3, we built a moderation model and found that the effects of social media influence and health consciousness on continuous usage intention were significant: beta=.124, *P=*.04. The respective effects of perceived information quality, perceived system quality, and health consciousness on continuous usage intention were insignificant. Thus, health consciousness partially moderated the impact of perceived information quality, perceived system quality, and social media influence on the continuous usage intention. Table 7 shows the results of the hypotheses testing.

**Table 7:** Results of the hypotheses testing.

Indicator (abbreviation)	Model I		Model II		Model III			Hypothesis test
	Path coefficient	*P* value	Path coefficient	*P* value		Path coefficient	*P* value	
Perceived information quality (PIQ)	.250	.001	.196	.003		.209	.003	Hypothesis 1 was supported
Perceived system quality (PSQ)	.205	.02	.179	.03		.178	.03	Hypothesis 2 was supported
Social media influence (SMI)	.302	<.001	.261	<.001		.254	<.001	Hypothesis 3 was supported
Attitude (AT)	.558	<.001	.558	<.001		.558	<.001	Hypothesis 5 was supported
Health consciousness (HC)	N/A^a^	N/A	.294	<.001		.269	<.001	N/A
PIQ × HC	N/A	N/A	N/A	N/A		.101	.17	Hypothesis 4a was not supported
PSQ × HC	N/A	N/A	N/A	N/A		.044	.56	Hypothesis 4b was not supported
SMI × HC	N/A	N/A	N/A	N/A		.124	.04	Hypothesis 4c was reverse supported
Age	.054	.25	.055	.24		.055	.25	N/A
Gender	.028	.62	.028	.60		.029	.60	N/A
Education	-.090	.28	.013	.83		.013	.83	N/A
Work	.013	.83	.090	.28		.090	.28	N/A
R^2^	.333	N/A	.411	N/A		.434	N/A	N/A
R^2^ adjusted	.325	N/A	.401	N/A		.418	N/A	N/A

^a^Not applicable.

## Discussion

### Principal Findings

This study examined the factors that affect the continuous usage intention of mHealth services based on the ELM and resulted in several findings. First, patients perceived mHealth services to have positive information quality; we also observed that the system quality of mHealth services had a positive effect on patients’ personal attitudes, indicating that when they perceived the provided mHealth apps to have a higher quality, they would change their attitudes. Our results are consistent with findings in prior studies [46]. Second, the results reveal that social media influence does have a positive effect on a patient’s attitude toward mHealth services, which is consistent with findings in existing research [47]. It indicates that effective social media influence can help patients improve their attitudes toward mHealth services.

Third, our study purports that patients’ health consciousness does moderate the influence of perceived information quality, perceived system quality, and social media on their attitudes. In particular, our findings suggest that a patient’s health consciousness has a positive effect on the relationship between social media influence and attitude, in contrast with Hypothesis 4c. This may be because patients with high health consciousness are more likely to pay attention to the management of personal health; this could lead them to discover new and effective health management modes and to try and continue to find more useful functions for health management, in order to manage their own health. However, patients with low health consciousness may not have a strong awareness of personal health management, and information about health promotion may be directly ignored. Boontarig also illustrated that the participant with a lower level of health consciousness is more concerned about value and convenience in supporting the use of the system. They also elaborated that the participant’s personality as a factor affecting a user’s perception is important in technology adoption and must be considered when developing and designing these services [48]. Thus, we infer that the effect of social media influence on patients’ attitudes toward mHealth services may be mediated through the patients’ health consciousness. Moreover, the moderation effects of health consciousness on the relationship between the perceived information quality of the content of mHealth services and the perceived system quality of mHealth services, as well as attitudes toward them, were found to be insignificant. This means that the patient’s health consciousness has no moderating effect on the central route (ie, perceived mHealth information quality and perceived mHealth system quality) and the patient’s attitude toward mHealth services. This may be because patients pay more attention to the quality of information and the system when using mHealth services and this has nothing to do with personal health consciousness. As long as the quality of mHealth services is verified, patients will choose to use mHealth services. Biduski at el illustrated that the determining aspect in user experience is whether the app features meet users’ health needs [49]. As long as the quality of mHealth services can meet the expectations of patients, patients will continue to use mHealth services.

### Theoretical Implications

To the best of our knowledge, this is the first study to use the ELM to explore the factors that affect a patient’s continuous usage intention on mHealth services based on multidimensional perspectives. Specifically, this study contributes to the mHealth service literature in three aspects.

First, in our research, we expanded the ELM and applied it to the mHealth care domain. Previous researchers have applied the ELM theory to many fields, including e-commerce, consumer behavior advertising, health care, and so on. Rollins and Bhutada illustrated that more highly involved consumers had more positive attitudes, behavioral intentions, and greater information-searching behavior [50]. Sher and Lee studied the effects of consumer skepticism on online shopping based on the ELM [51]. Angst and Agarwal also found that privacy concerns plays a significant role in the adoption of electronic health records [52]. Based on the ELM, this study explained the mechanism of influencing patients’ continuous usage intention toward mHealth services.

Second, we explored the central and peripheral routes, with their different motivations and abilities and their effects on user behavior. The central route (ie, perceived mHealth information quality and perceived mHealth system quality) is the factor that patients are most concerned with, which directly affect whether patients accept the mHealth services mode. As well, the peripheral route (ie, social media influence) can affect patients’ attitudes toward mHealth services through effective communication channels, such as TV shows, magazines, or advertisements, then change their continuous usage intention of mHealth services. However, due to individual differences (ie, patients’ health consciousness), the process and response of these things are not the same, so patients’ health consciousness plays a significant role between the central route, the peripheral route, and patients’ attitudes toward mHealth services.

Third, we redefined the cognitive-attitude-change process based on the original ELM. The continuous usage behavior of mHealth services needs to go through two stages: the first stage is a process from cognitive-to-attitude change and the second stage is a process from the attitude change to continuous usage, which can be well explained by the ELM theory.

### Practical Implications

Our research provides relevant practical implications. First, through the verification of this model, hospital administrators are provided with theoretical support, which enables them to better grasp the patient’s psychological characteristics and make their information construction more comprehensive in the process of informatization. Second, for health management entrepreneurs, this model can provide support for the design of health management apps; it can help entrepreneurs clearly identify the problems they encounter and help them decide which directions to take. Third, the patients themselves can gain a clear understanding of their inner tendencies so they can identify health management systems that suit their characteristics within many health management apps, in order to better manage their own health care.

### Limitations and Directions for Future Research

Some limitations of this study need to be considered. First, our data was collected through online questionnaires. Although the participants were from all over the country, and though they were representatives of each province, there were also certain limitations. In a future field study, we will need to use a health management software app designed by our laboratory so that we can measure the users’ actual usage. Second, we measured continuous usage intention rather than actual usage. Although many theories, such as the theory of reasoned action [53] and the theory of planned behavior [54], and empirical studies have shown that behavioral intention is a reliable proxy for actual human behavior, future studies need to explore how and which factors influence actual usage behavior. Finally, we tried to conduct group experiments for health consciousness—high and low groups—to test whether there were different effects on continuous usage intention when patients experienced different degrees of health consciousness, but because of data constraints, the results did not differ significantly. Hence, we will measure and analyze the effects of this variable in the future.

### Conclusions

This study explored the impacts of mHealth services on patient’s continuous usage intention from three dimensions, including personal intrinsic factors, organizational factors, and social factors. We found the following: (1) the influence of the individual’s internal motivation on his or her health behavior is positively correlated, (2) the influence of external factors, such as social media, on a patient’s health behavior is positive, and (3) a patient’s health consciousness plays a positive role in changing a person’s health continuous usage behavior. The findings of this article are important contributions to the field for both scholars and practitioners.

## Appendix

Multimedia Appendix 1 Measurement items.

## Abbreviations

AMOS: analysis of moment structure
ELM: elaboration-likelihood model
EQS: equation structural program
IT: information technology
LISREL: linear structural relationships
mHealth: mobile health
PLS: partial least squares
SEM: structural equation modeling
