# Supplementary material for: Exploring Patients' Intentions for Continuous Usage of mHealth Services: Elaboration-Likelihood Perspective Study
Source: JMIR Mhealth Uhealth. 2020 Apr 6;8(4):e17258. doi: 10.2196/17258 (PMC7171561; doi:10.2196/17258)
Supplement: Multimedia Appendix 1 [file mhealth_v8i4e17258_app1.docx]

# Exploring Patients' Intentions for Continuous Usage of mHealth Services: Elaboration-Likelihood Perspective Study

**Appendix**

*Measurement Items*

| Construct Name and Definition | Coding | Item Type | Item | Source |
| --- | --- | --- | --- | --- |
| Dependent Variables | | | | |
| Perceived mHealth Information Quality | PCQ1 | Original Item | I think that e-learning will provide various forms of information. | (Park et al., 2005) |
|  |  | Adapted Item | I think that the mHealth management APP will provide ample information. |  |
|  | PCQ2 | Original Item | I think that the information I will obtain from e-learning is valuable. |  |
|  |  | Adapted Item | I think that the mHealth management APP will provide valuable information. |  |
|  | PCQ3 | Original Item | E-learning will provide the information and services I need. |  |
|  |  | Adapted Item | I think that the mHealth management APP will provide needy information. |  |
|  | PCQ4 | Original Item | The information provided during the DMS training session was persuasive. | (Bhattacherjee & Sanford, 2006) |
|  |  | Adapted Item | I think that the mHealth management APP will provide persuasive information. |  |
| Perceived mHealth Service Quality | PSQ1 | Original Item | I think that the e-learning APP will provide a very reliable service. | (Park et al., 2005) |
|  |  | Adapted Item | I think that the mHealth management APP will provide a very reliable service |  |
|  | PSQ2 | Original Item | I would say that XYZ provides superior service. | (Brady & Jr, 2001) |
|  |  | Adapted Item | I would say that the mHealth management APP provides superior service. |  |
|  | PSQ3 | Original Item | I believe that XYZ offers excellent service. |  |
|  |  | Adapted Item | I believe the mHealth management APP offers excellent service. |  |
| Social Media Influence | SMS1 | Original Item | The magazines I read and the TV shows I watch emphasize that it is important to be thin. | (Keery, Van, & Thompson, 2004) |
|  |  | Adapted Item | The magazines I read and the TV shows I watch emphasize that it is important to be healthy. |  |
|  | SMS2 | Original Item | The magazines I read and the TV shows I watch emphasize the importance of appearance (shape, weight, clothing). |  |
|  |  | Adapted Item | The magazines I read and the TV shows I watch emphasize the importance of being healthy. |  |
|  | SMS3 | Original Item | The magazines I read and the TV shows I watch emphasize dieting to lose weight. |  |
|  |  | Adapted Item | The magazines I read and the TV shows I watch emphasize keeping healthy through good habits. |  |
| Attitude | AT1 | Original Item | I (dislike/like) the idea of using the system. | (Kim, 2009) |
|  |  | Adapted Item | I like the idea of using this mHealth management APP |  |
|  | AT2 | Original Item | Using a spreadsheet software makes me feel good. | (Yang & Yoo, 2005) |
|  |  | Adapted Item | Using this mHealth management APP would make me feel good. |  |
|  | AT3 | Original Item | Using a spreadsheet software makes me feel positive. |  |
|  |  | Adapted Item | Using this mHealth management APP would make me feel positive. |  |
|  | AT4 | Original Item | A spreadsheet is a helpful instrument for performing my tasks |  |
|  |  | Adapted Item | This mHealth management APP is a helpful instrument for protecting my health. |  |
|  | AT5 | Original Item | A spreadsheet is a beneficial instrument for performing my tasks |  |
|  |  | Adapted Item | This mHealth management APP is a beneficial instrument for protecting my health. |  |
|  | AT6 | Original Item | A spreadsheet is a valuable instrument for performing my tasks |  |
|  |  | Adapted Item | This mHealth management APP is a valuable instrument for protecting my health. |  |
| Independent Variables | | | | |
| Continuous Usage Intention | CUI1 | Original Item | I intend to use DMS for more of my job responsibilities. | (Bhattacherjee & Sanford, 2006) |
|  |  | Adapted Item | I intend to continue using mHealth mangement APP for more of my health management responsibilities. |  |
|  | CUI 2 | Original Item | I intend to use anti-spyware software in the next three months | (Johnston & Warkentin, 2010) |
|  |  | Adapted Item | I intend to continue using mHealth mangement APP in the next three months. |  |
|  | CUI 3 | Original Item | I predict I will use the anti-spyware software in the next three months. |  |
|  |  | Adapted Item | I predict I will continue using mHealth mangement APP in the next three months. |  |
|  | CUI 4 | Original Item | I plan to use the anti-spyware software in the next three months. |  |
|  |  | Adapted Item | I plan to continue using mHealth mangement APP in the next three months. |  |
| Moderator Variables | | | | |
| Health Consciousness | HC1 | Original Item | I reflect about my health a lot. | (Mai & Eisenberg, 2012) |
|  | HC2 | Original Item | I am very self-conscious about my health. |  |
|  | HC3 | Original Item | I am generally attentive to my inner feelings about my health. |  |
|  | HC4 | Original Item | I am constantly examining my health conditions. |  |
|  | HC5 | Original Item | I think that I take health matters into account a lot in my life. | (Chen, 2008) |
|  | HC6 | Original Item | I think it is important to know well how to eat healthily. |  |
